# The impact of height on the spread of spinal anesthesia and stress response in parturients undergoing caesarean section: a prospective observational study

**DOI:** 10.1186/s12871-021-01523-2

**Published:** 2021-11-30

**Authors:** Ying-Jun She, Wen-Xing Liu, Ling-Yu Wang, Xin-Xu Ou, Hui-Hong Liang, Dong-Xu Lei

**Affiliations:** Department of Anesthesiology and Perioperative Medicine, Guangzhou Women and Children’s Medical Center, Guangzhou Medical University, 9# Jinsui Road, Tianhe district, Guangzhou, 510623 China

**Keywords:** Body height, Spinal, Amniotic fluid index, Cesarean section

## Abstract

**Background:**

The spread of spinal anesthesia was influenced by many factors, and the effect of body height on spinal anesthesia is still arguable. This study aimed to explore the impact of height on the spread of spinal anesthesia and the stress response in parturients.

**Methods:**

A total of ninety-seven parturients were allocated into two groups according to their height: the shorter group (body height was shorter than 158 cm) and taller group (body height was taller than 165 cm). Spinal anesthesia was performed with the same amount of 12 mg plain ropivacaine in mothers of different heights. The primary outcome of the study was the success or failure of the spinal anesthesia. The secondary outcomes of the study were stress response, time to T6 sensory level, the incidence of hypotension, the satisfaction of abdominal muscle relaxation and patient VAS scores.

**Results:**

The rate of successful spinal anesthesia in the shorter group was significantly higher than that in the taller group (*p* = 0.02). The increase of maternal cortisol level in the shorter group was lower than that in the taller group at skin closure (*p* = 0.001). The incidence of hypotension (*p* = 0.013), time to T6 sensory block (*p* = 0.005), the quality of abdominal muscle relaxation (*p* <  0.001), and VAS values in stretching abdominal muscles and uterine exteriorization (*p* <  0.001) in the shorter group were significantly different from those in the taller group. Multivariate analysis showed that vertebral column length (*p* <  0.001), abdominal girth (*p* = 0.022), amniotic fluid index (*p* = 0.022) were significantly associated with successful spinal anesthesia.

**Conclusions:**

It’s difficult to use a single factor to predict the spread of spinal anesthesia. Patient’s vertebral column length, amniotic fluid index and abdominal girth were the high determinant factors for predicting the spread of spinal anesthesia.

**Trials registration:**

ChiCTR-ROC-17012030 (Chictr.org.cn), registered on 18/07/2017.

## Introduction

Spinal anesthesia is the preferred choice of anesthesia for cesarean section with its reliable and rapid effect. And it has many advantages over general anesthesia because it provides effective postoperative pain relief, keeps the parturients conscious of the delivery, and minimizes the chance of maternal aspiration and difficult intubation. A large variable clinical dosage was used for spinal anesthesia in pregnant women with cesarean section [1]. Spinal injection of a local anesthetic usually produces unpredictable levels and durations of anesthesia. Higher cephalad spread of spinal anesthesia may lead to excessive sympathetic nerve depression such as respiratory depression and severe hypotension in pregnant women. Maternal hypotension leads to a reduction in maternal and uteroplacental blood perfusion and develops the potential for fetal acidemia. Inadequate spread of spinal anesthesia may provide the stability of breathing and circulation, but it may bring intraoperative pain and inadequate degree of anesthesia.

The spread of spinal anesthesia was influenced mainly by many factors including age [2], height [3, 4], weight [5, 6], body mass index [7, 8], abdominal girth [9, 10], vertebral column length [9, 11], the baricity of the injected solution [12, 13], patient position [13] and the volume of lumbosacral cerebrospinal fluid (CSF) [14, 15]. The volume of lumbar CSF is thought to be the most important factor for the extent of spinal anesthesia. The decrease in its volume leads to an increase in the concentration of local anesthetic, which produces a broader blockade. However, it’s impractical to measure the volume and pressure of lumbar CSF during perioperative period. Patient general characteristics such as body mass index, weight and height are often used to determine the spread of spinal anesthesia because they are easy to measure. Some studies suggest that the spread of spinal anesthesia tends to increase with increasing weight and decrease linearly with height [16, 17], but more studies have taken the opposite view, suggesting that weight is independently associated with the outcomes of spinal anesthesia [13, 18, 19]. And the effect of body height on spinal anesthesia is still arguable, with inconsistent results reported in the literature [3, 4, 9, 20–22]. Our previous study showed no significant difference in spinal ropivacaine dose requirement between taller and shorter pregnant women [21]. However, the limitation of this study was that the difference in average height between the taller and shorter groups was small, and this may influence the spinal dosage in pregnant women with more significant height differences. Hence, it’s necessary to further explore the application of spinal anesthesia in the pregnant population with a more considerable average height difference.

Spinal ropivacaine has been effectively and safely used for obstetric patients, with some advantages such as lower central nervous and cardiac toxic potential. The aim of this study was to compare the effect of the same dose of spinal ropivacaine on the spread of spinal anesthesia and stress response in people of different heights, and also evaluate the determinant factors for predicting the spread of spinal anesthesia. We hypothesized that the spread of spinal anesthesia produced by the same dose of ropivacaine in shorter pregnant women was greater than that in taller pregnant women.

## Methods

This study was approved by the Institutional Review Board of Guangzhou Women and Children’s Medical Center (IRB2013007), Guangzhou, China. The trial was registered prior to patient enrollment at chictr.org.cn (ChiCTR-ROC-17012030, registered on 18/07/2017). This study was conducted from August 2017 to July 2019 in accordance to Good Clinical Practice guidelines and the Declaration of Helsinki for experiments involving humans. Written informed consent was obtained from all eligible parturients. Ninety-seven ASA physical status I or II parturients scheduled for elective cesarean delivery from 8 am to noon were recruited into the study. Exclusion criteria included age younger than 18 years or older than 40 years, congenital anomaly, ruptured membranes, placenta praevia, cardiovascular, cerebrovascular or renal disease, bleeding disorders, infection at the site of injections, gestational age < 37 weeks, known abnormal fetal development, treatment with drugs known to influence anesthetic requirement or allergy and other conditions that were considered unsuitable for this study by the attending anesthesiologists. After enrolment, parturients were allocated into two groups according to their height: shorter group (Group S, body height was shorter than 158 cm) and taller group (Group T, body height was taller than 165 cm). The cut-off point for body height was based on both the characteristics of parturients height parameters in Chinese city and our department’s data derived from our previous study [21, 23].

All parturients underwent preoperative fasting for 8 h and did not have any premedication. A 20-G IV catheter was placed in a peripheral vein in the parturient’s forearm, and a loading infusion of lactated Ringer’s solution 500 ml was administrated before spinal anesthesia. After entering the operating room, standard monitoring such as non-invasive blood pressure measurement, pulse oximetry and electrocardiography was attached, the baseline was recorded. All patients received combined spinal-epidural anesthesia in the left lateral position. Epidural/spinal puncture were performed at the L3–4 interspace after the skin was infiltrated with 1% lidocaine, and the spinal component was performed using a 27 G pencil-point needle via a needle-through-needle technique. The spinal local solutions were prepared by mixing 1% plain ropivacaine 1.2 mL (12 mg) with cerebrospinal fluid to make a total volume of 2.5 mL in all cases. The spinal solutions were injected into the subarachnoid space in 10 s. After an epidural catheter was placed, the patient was positioned at a 15°left lateral tilt. Non-invasive arterial pressure was monitored every 2 min for the first 20 min after spinal injection, and then every 5 min throughout surgery. Surgery was started when the sensory block was higher than T6 dermatome level. Oxygen was administered through a face mask at a flow rate of 3 L/min during the operation. All spinal anesthesia procedures were operated by the same anesthesiologist. Then another anesthesiologist, who was blind to the group assignment and parturient’s measurements, was responsible for outcome evaluations after completion of spinal anesthesia procedures.

The sensory level of spinal anesthesia was assessed bilaterally at the midclavicular line by pinprick test with 2 min intervals for the first 20 min after drug administration, and assessed at 25 min, and then assessed with 15 min intervals until the completion of surgery. Onset time to T6 was defined as the time from the spinal injection to loss of pinprick sensation to T6 level. The time to reach bilateral sensory block at the T6 level was recorded. The highest sensory level of spinal block and dermatome level at both incision and surgery completion were collected. The quality of abdominal muscle relaxation was scaled by the surgeon after completion of surgery as follow: 1 = excellent (muscles are very relaxed), 2 = good (muscles are relaxed), 3 = neutral (disturbing, but acceptable muscle strain), 4 = unsatisfactory (muscles are strain), 5 = poor (muscles are very strain, unacceptable). The basic demographic parameters, including age, weight, height, parity, weight gain during pregnancy, times of previous cesarean, and gestational age were collected by an independent nurse on enrollment in the study. Neonate weight and Apgar scores at 1 min and 5 min were collected after delivery. The body mass index (BMI) value of each pregnant woman was calculated by the collected height and weight in the study. The amniotic fluid index was obtained from the color doppler ultrasound within 3 days before the operation. If the pregnant woman did not have color doppler ultrasound within 3 days before the operation, this case was excluded from this study. The vertebral column length was defined from the sacral hiatus to the seventh cervical vertebra after the parturient was in the lateral position with their neck, back and legs flexed. The lowest and highest values of heart rate, systolic and diastolic blood pressure were all recorded. Visual analogue scale (VAS) score, which is from 0 (no pain) to 100 (the most severe pain), was used to assess the pain during the operation at four time points: skin incision, delivery, uterine exteriorization, and skin closure. The abdominal girth was measured at the level of the umbilicus in the supine position during the end of expiration by the same investigator. These data were not revealed to the anesthesiologist both performing the procedure and assessing spinal blocks.

The primary outcome of the study was the success or failure of the spinal anesthesia. Successful anesthesia was defined as a bilateral sensory level to pinprick to T6 level within 15 min and no additional epidural local anesthetic required during surgery. When the T6 sensory level was not reached within 15 min after spinal injection, or when the parturient requested additional analgesia during surgery, or when VAS score was greater than 40 mm, epidural anesthesia with 2% lidocaine 5 ml was used to enhance intraoperative pain control and repeated as required. The secondary outcomes of the study were stress response, time to T6 sensory level, the incidence of hypotension, the satisfaction of abdominal muscle relaxation, patient VAS scores at skin incision, stretching abdominal muscles, uterine exteriorization, and skin closure. Side effects included nausea and vomiting, hypotension, bradycardia, shivering, and others.

Hypotension was defined as systolic blood pressure lower than 90 mmHg or a decrease larger than 30% from baseline and was treated with intravenous ephedrine 6 mg. Bradycardia was defined as heart rate lower than 50 beats/min and treat with 0.5 mg atropine intravenously. The baseline heart rate and arterial blood pressure were measured by calculating the average values of three consecutive readings at 2 min intervals. Stress hormones responses were assessed by determining plasma epinephrine, norepinephrine, dopamine and cortisol via enzyme immunoassay. Blood samples were collected from lower limb vein of the parturients at the following intervals: before skin incision and skin closure. Blood samples were also collected from the umbilical vein to measure stress response at delivery.

### Statistical analysis

All results are expressed as mean (SD) or median (range) as appropriate. Statistical analyses were determined using SPSS version 25.0 for Windows (Chicago, IL). With a type one error of 5% and a power of 90%, 86 subjects were required to detect a difference in the incidence of successful spinal anesthesia with a significance level of 0.05. This hypothesis was based on the data from our pilot study, in which there was a 25% difference in the success rate of spinal anesthesia between shorter and taller groups (65% in taller group and 90% in shorter group). While considering the dropout rate of about 5%, the final sample size was determined to be 90 subjects. Data were assessed for normal distribution of variance using the Shapiro-Wilks test. Means were assessed by one way analysis of variance. Medians and non-normally distributed means were assessed by Mann-Whitney U test. Incidence data were analyzed by the chi-squared test or Fisher’s exact test. A binary logistic regression was used to determine the correlation of successful spinal anesthesia and patient’s characteristics. A *p*-value < 0.05 were considered statistically significant.

## Results

**Fig. 1:**
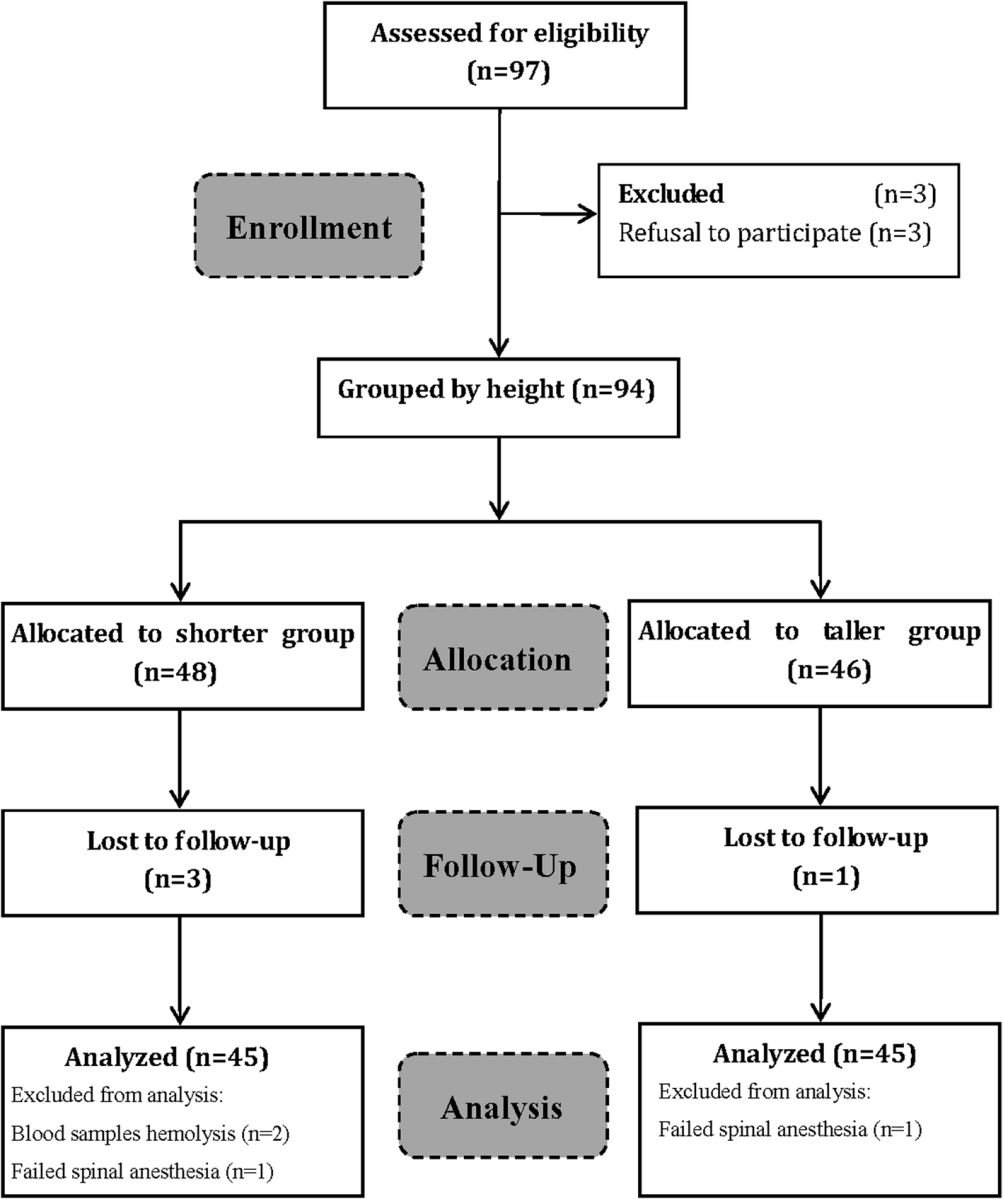
Flow diagram outlining the study procedure

A total of 90 recruited parturients completed the study without any protocol violations. Seven patients were excluded. Two patients were excluded because of failed spinal anesthesia, two patients were excluded due to hemolysis in blood samples, and three were excluded because they declined to participate in the study (Fig. 1). The characteristics of neonates and parturients were presented in Table 1. There were no baseline differences between the groups regarding age, parity, number of pregnancy, gestation week, duration of surgery, Apgar scores at 1 and 5 min, and neonatal weight. However, the demographic data including height, weight, BMI, vertebral column length, abdominal girth, amniotic fluid index and weight gain in the shorter group were significant difference compared with these in the taller group.

**Table 1:** Patient characteristics and clinical parameters

items	Group S	Group T	*P* values
Number of pregnancy	2.3 ± 1.0	2.4 ± 0.8	0.429
Parity	0.9 ± 0.5	0.9 ± 0.6	0.861
Age (years)	33.2 ± 3.9	32.7 ± 4.9	0.584
Weight (kg)	63.7 ± 8.0	72.7 ± 6.8	< 0.001
Height (cm)	153.1 ± 3.5	169.5 ± 2.1	< 0.001
Body mass index (kg/m^2^)	27.2 ± 3.1	25.3 ± 2.3	0.002
Vertebral column length (cm)	60.7 ± 3.0	68.0 ± 3.4	< 0.001
Gestation (wk)	38.4 ± 0.8	38.5 ± 0.8	0.793
Abdominal girth (cm)	96.1 ± 5.1	98.0 ± 5.0	0.09
Amniotic fluid index (mm)	150.4 ± 44.2	137.3 ± 25.2	0.09
Weight gain (kg)	12.6 ± 3.5	14.5 ± 3.8	0.017
Duration of surgery (min)	42.5 ± 13.4	41.8 ± 13.2	0.813
Apgar score at 1 min	9.5 ± 0.5	9.5 ± 0.5	0.701
Apgar score at 5 min	9.9 ± 0.3	9.9 ± 0.3	0.508
Neonatal weight (kg)	3.2 ± 0.5	3.3 ± 0.4	0.113
Dermatome level at incision	T5	T5	0.767
Highest level of block	T3	T4	0.002
Dermatome level at surgery completion	T7	T7	0.755

Values are mean ± SD or number. Group S: shorter group; Group T: taller group

Forty out of 45 patients experienced successful spinal anesthesia in the shorter group, and thirty one out of 45 patients had successful spinal anesthesia in the taller group. There was a significant difference in the rate of successful spinal anesthesia between the shorter and taller groups (*p* = 0.02). The incidence of hypotension (*p* = 0.013), time to T6 sensory block (*p* = 0.005), the effect and quality of abdominal muscle relaxation (*p* <  0.001), and the VAS values in both stretching abdominal muscles and uterine exteriorization (*p* <  0.001) in the shorter group were significantly different from those in the taller group, which are summarized in Table 2. In the multivariate model with successful spinal anesthesia as the outcome, vertebral column length (*p* <  0.001), abdominal girth (*p* = 0.022), amniotic fluid index (*p* = 0.022) were significantly associated with successful spinal anesthesia, whereas height, weight, BMI and weight gain were not related (Table 3).

**Table 2:** Characteristics of spinal anesthesia and adverse events

Items	Group S	Group T	*P* values
Time to T6 sensory block (min)	6.6 ± 2.1	8.0 ± 2.3	0.005
Successful spinal anesthesia (n)	40 (89)	31 (69)	0.02
Hypotension (n)	23 (51)	12 (27)	0.013
Abdominal muscles tone scores	2.1 ± 1.0	3.0 ± 1.0	< 0.001
VAS values			
Skin incision	1.4 ± 0.5	1.5 ± 0.6	0.700
Stretching abdominal muscles	2.3 ± 1.1	3.2 ± 1.1	< 0.001
Uterine exteriorization	2.2 ± 0.6	3.0 ± 0.8	< 0.001
Skin closure	1.6 ± 0.5	1.6 ± 0.5	0.685
Nausea and vomiting (n)	19 (42)	16 (36)	0.333
Ephedrine requirement (mg)	3.5 ± 3.7	2.0 ± 3.5	0.035

Values are mean ± SD or number (%). Group S: shorter group; Group T: taller group

**Table 3:** Factors associated with the success of spinal anesthesia

Variables	Univariate analysis		Multivariate analysis	
	Odds ratio (95% CI)	*P*	Odds ratio (95% CI)	*P*
Vertebral column length	0.77 (0.66–0.89)	< 0.001	0.73 (0.61–0.85)	< 0.001
Abdominal girth	1.13 (1.00–1.28)	0.049	1.25 (1.03–1.52)	0.022
Amniotic fluid index	1.03 (1.01–1.05)	0.007	1.03 (1.01–1.06)	0.022
Height	0.88 (0.81–0.96)	0.003	0.97 (0.83–1.15)	0.74
Weight	0.92 (0.85–0.98)	0.011	1.48 (0.46–4.82)	0.511
Body mass index	1.00 (0.83–1.20)	0.99	0.82 (0.62–1.08)	0.152
Weight gain	1.00 (0.87–1.14)	0.97	1.10 (0.87–1.39)	0.422

Values are Odds Ratio (95% confidence intervals)

**Fig. 2:**
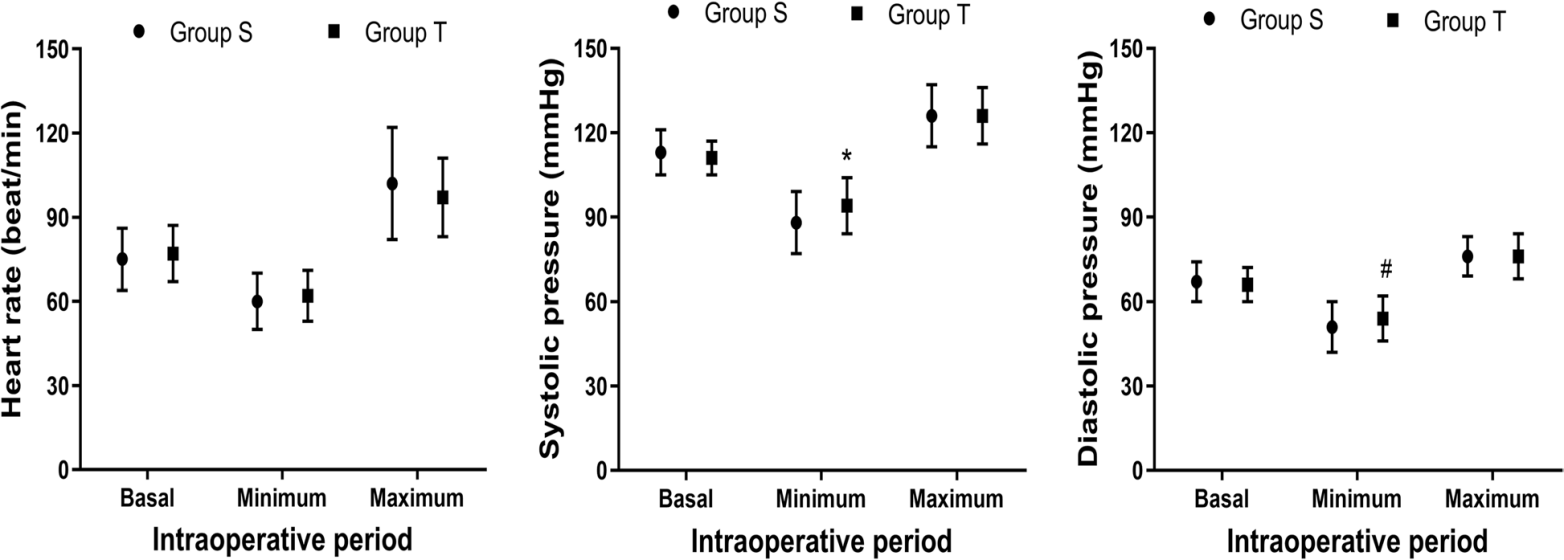
Hemodynamic data including heart rate and blood pressure in both groups. There were no differences between the shorter and taller groups concerning the basal level of heart rate, systolic pressure and diastolic pressure. Compared with Group S, * *p* = 0.039, # *p* = 0.013. Group S: shorter group; Group T: taller group

Hemodynamic data was presented in Fig. 2. There were no differences between the shorter and taller groups concerning the basal level of heart rate, systolic pressure and diastolic pressure. The minimum values of diastolic (*p* = 0.013) and systolic (*p* = 0.039) pressure in the taller group were significantly higher than those in the shorter group. The median dose of administered ephedrine in the shorter group was larger than that in the taller group (*p* = 0.035).

**Fig. 3:**
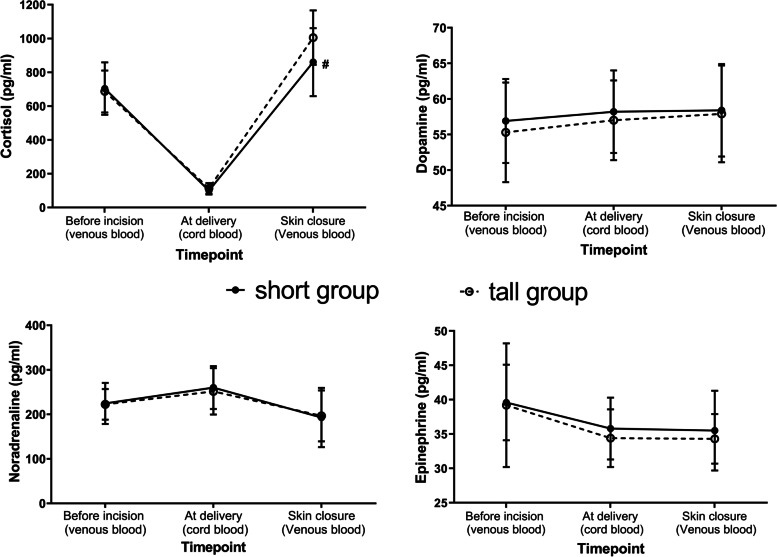
Stress responses in both groups. There were no significant differences between the shorter and taller groups concerning the basal level of plasma epinephrine, norepinephrine, dopamine and cortisol. Compared with Group S, # *p* = 0.001. Group S: shorter group; Group T: taller group

There were no significant differences between the shorter and taller groups concerning the basal level of plasma epinephrine, norepinephrine, dopamine and cortisol (Fig. 3). For maternal cortisol levels, the shorter group had an increase to 122% of the pre-incision value at end of surgery, and the taller group had an increase to 146% of pre-value at skin closure; and maternal cortisol level in shorter group was lower than that in taller group at skin closure (*p* = 0.001). The cortisol level in cord blood was significantly lower than that in venous blood. There was no difference between the shorter and taller groups in postoperative epinephrine, norepinephrine and dopamine in both maternal venous and cord blood.

## Discussion

In this prospective observational study, a comparison of the parameters related to spinal anesthesia in pregnant women grouped by short and tall body height was performed to determine the effect of body height on the outcome of spinal anesthesia. The incidence of successful spinal anesthesia and hypotension was significantly higher in shorter patients. Onset time to T6 level in shorter patients was significantly faster compared with taller patients. The effect and quality of abdominal muscle relaxation and VAS values in both stretching abdominal muscles and uterine exteriorization in the shorter group were greater than those in the taller group. A decrease in stress hormone levels in the shorter group was significantly induced compared with taller group.

Surgical stress response induces a predictable cascade of metabolic, immunologic and endocrine responses through activation of somatic and sympathetic nervous systems. The level of plasma cortisol increases with the intensity of stress, so it was usually used as a marker of stress response [24]. Pain relief could suppress the release of stress hormones during surgery [25]. Spinal and epidural anesthesia have been shown to reduce the maternal stress response during cesarean section compared with general anesthesia [26, 27]. In the present study, our results indicated that the increase of maternal cortisol level in the taller group was significantly greater than that in the shorter group at end of surgery. And this suggested that patients in shorter group had better pain control and fewer stress response induced by surgical trauma than those in the taller group. Although maternal stress response was affected, the stress response in the fetus was not influenced by the use of spinal anesthesia in pregnant women with different body height in our study. This is similar to previous findings that fetal stress response is not affected by the type of anesthesia [27].

Beside maternal cortisol, our results showed that patients in the shorter group had more satisfactory anesthesia including higher incidence of successful spinal anesthesia, greater abdominal muscle relaxation and better pain control in both stretching abdominal muscles and uterine exteriorization. To the best of our knowledge, there has been no study comparing the effect of spinal anesthesia in terms of only maternal height during cesarean section. Some previous similar studies have shown that small dose local anesthetics adjusted according to height and weight can provide satisfactory spinal anesthesia [3, 22]. Patient’s height was identified to be a risk factor for maternal hypotension [3, 4]. Also, the shorter group was associated with faster onset time to T6 level and a higher incidence of hypotension. And this suggested that the shorter group has a faster spread of spinal anesthesia, which were similar to some studies and different from Norris report [1, 4, 20, 21]. However, in addition to height, there were significant differences in weight, body mass index, vertebral column length, abdominal girth, amniotic fluid index and weight gain among the basic parameters of patients in the two groups grouped by height in our study. Therefore, the higher rate of successful spinal anesthesia, faster reaching T6 level, better abdominal muscle relaxation and lower stress response in the shorter group could not be attributed solely to height. We intended to use only one variable and divide patients into two groups based on their height in our study. However, taller patients are more likely to be heavier and have longer spines than shorter patients. It is almost impossible for all parameters other than height to be similar in the two groups grouped by height in practice. Further exploration with multivariate analysis in our study showed that vertebral column length, amniotic fluid index and abdominal girth, but not height, were significantly associated with spinal anesthesia success.

In this study, the success rate of spinal anesthesia was 69 and 89% in taller and shorter groups, respectively. In addition to vertebral column length, amniotic fluid index, abdominal girth and the volume of lumbar cerebrospinal fluid, there are many other factors that affect the success rate of spinal anesthesia in pregnant women. In this study, plain ropivacaine was used for spinal injection in mothers of different heights. As reported by Fettes et al. hyperbaric solutions of ropivacaine by addition of glucose produced greater spinal block and onset time than plain solutions of ropivacaine in patients with spinal anesthesia [12]. The dose of ropivacaine was also an important factor, and the 12 mg ropivacaine used in this study was smaller than the 15 mg reported by Fettes et al. However, the 12 mg dose of ropivacaine is the usual dose of spinal solutions in our department, which can provide satisfactory analgesia and smooth hemodynamics for most pregnant women. In addition, the size of spinal anesthesia needles and the different criteria which define the success of spinal anesthesia also affect the success rate of spinal anesthesia [28].

The volume of lumbosacral CSF has been revealed as the major determinant for the spinal spread and duration. Larger abdominal girth is positively associated with an increase in intra-abdominal pressure which could decrease the lumbosacral CSF volume and extent spread of spinal anesthesia [29]. Zhou et al. found that there was a strong correlation between abdominal girth and lumbosacral CSF volume measured by magnetic resonance imaging [30]. It has been showed that abdominal girth was correlated with maximal spread of spinal anesthesia [31]. Some previous studies have been consistent with our view that abdominal girth could be an easy observable factor in predicting the spread of spinal anesthesia [9, 11, 19]. Vertebral column length was another key determinant of the spread of spinal anesthesia, and this result was consistent with most other studies [9, 11, 19, 32, 33]. Hocking and Wildsmith suggested that the difference in height between adults is mainly the difference in the length of the lower limbs, rather than the difference in vertebral column length [13]. A survey on the height of Chinese pregnant women divided the subjects into three different body height groups. The results showed that there was a correlation between body height and vertebral column length, but the correlation coefficient of each group was small. And there was no significant difference in vertebral column length among the three different body height groups [34]. In this study, we enlarged the body height difference between the two groups to 16.4 cm, while the difference in vertebral column length was 7.3 cm. Interestingly, our results showed that a 7.3 difference in vertebral column length, but not a 16.4 cm difference in body height, was an important factor affecting the spread of spinal anesthesia. Importantly, vertebral column length is considered to be positively correlated with lumbosacral CSF volume, thereby affecting the concentration of local anesthetics to change the degree of spinal anesthesia [30].

Amniotic fluid index is another important indicator of affecting spinal anesthesia success in our study. During pregnancy, amniotic fluid surrounds the fetus and provides a protective and supportive environment for fetal growth and development. Amniotic fluid index, which is summarized as the four-quadrant sum technique, is the most commonly employed techniques for measuring amniotic fluid volume [35, 36]. The normal amniotic fluid index is between 8 and 25 cm, and amniotic fluid index more than 25 was defined as polyhydramnios [36]. There was a positive correlation between uterine size and amniotic fluid volume, and thus a positive correlation between high amniotic fluid index and uterine volume [37, 38]. The enlarged gravid uterus constricts the inferior vena cava, causing epidural venous plexus to dilate and subsequently reduce the lumbar cerebrospinal fluid [39–41]. These could explain the higher success rate of spinal anesthesia in the shorter group with higher amniotic fluid index.

There were two limitations in the present study. First, the lumbosacral CSF volume can be estimated by magnetic resonance examination, the correlation between lumbosacral CSF volume and three determinant factors (vertebral column length, amniotic fluid index and abdominal girth) were not evaluated in our study. Some previous studies have found a positive correlation between two determinant factors (vertebral column length and abdominal girth) and lumbosacral CSF volume [30]. Further studies should be performed to determine the correlation between amniotic fluid index and lumbosacral CSF volume. Second, the combination of spinal and epidural anesthesia was used in our study, which may partly influence the outcomes.

## Conclusions

This study suggested that it’s difficult to use a single factor to predict the spread of spinal anesthesia. Our results indicated that the combination of patient’s vertebral column length, amniotic fluid index and abdominal girth, but not height, were the critical determinant factors for predicting the spread of spinal anesthesia. Patients with shorter vertebral column length, larger amniotic fluid index and abdominal girth have greater cephalad spread after spinal ropivacaine injection.

## Data Availability

The datasets are not publicly available, but available from the corresponding author on reasonable request.

## Abbreviations

CSF: Cerebrospinal fluid
BMI: Body mass index
VAS: Visual analogue scale
